# Evaluating complex health financing interventions: using mixed methods to inform further implementation of a novel PBI intervention in rural Malawi

**DOI:** 10.1186/s12913-016-1612-2

**Published:** 2016-08-19

**Authors:** Shannon A. McMahon, Stephan Brenner, Julia Lohmann, Christopher Makwero, Aleksandra Torbica, Don P. Mathanga, Adamson S. Muula, Manuela De Allegri

**Affiliations:** 1 Institute of Public Health, Faculty of Medicine, Heidelberg University, INF 324, 69120 Heidelberg, Germany; 2 Department of Public Health, University of Malawi, College of Medicine, Blantyre, Malawi; 3 Department of Policy Analysis and Public Management, Centre for Research on Health and Social Care Management (CERGAS), Bocconi University, Milano, Italy

**Keywords:** Performance-based incentives, Performance-based financing, Health financing, Results based financing, Process evaluation, Malawi, Research protocol

## Abstract

**Background:**

Gaps remain in understanding how performance-based incentive (PBI) programs affect quality of care and service quantity, whether programs are cost effective and how programs could be tailored to meet client and provider needs while remaining operationally viable. In 2014, Malawi’s Ministry of Health launched the Service Delivery Integration-PBI (SSDI-PBI) program. The program is unique in that no portion of performance bonuses are paid to individual health workers, and it shifts responsibility for infrastructure and equipment procurement from facility staff to implementing partners. This protocol outlines an approach that analyzes processes and outcomes, considers expected and unexpected consequences of the program and frames the program’s outputs relative to its costs. Findings from this evaluation will inform the intended future scale-up of PBI in Malawi.

**Methods/design:**

This study employs a prospective controlled before-and-after triangulation design to assess effects of the PBI program by analyzing quantitative and qualitative data from intervention and control facilities. Guided by a theoretical framework, the evaluation consists of four main components: service provision, health worker motivation, implementation processes and costing. Quality and access outcomes are assessed along four dimensions: (1) structural elements (related to equipment, drugs, staff); (2) process elements (providers’ compliance with standards); (3) outputs (service utilization); (4) experiential elements (experiences of service delivery). The costing component includes costs related to start-up, ongoing management, and the cost of incentives themselves. The cost analysis considers costs incurred within the Ministry of Health, funders, and the implementing agency. The evaluation relies on primary data (including interviews and surveys) and secondary data (including costing and health management information system data).

**Discussion:**

Through the lens of a PBI program, we illustrate how complex interventions can be evaluated via not only primary, mixed-methods data collection, but also through a wealth of secondary data from program implementers (including monitoring, evaluation and financial data), and the health system (including service utilization and service readiness data). We also highlight the importance of crafting a theory and using theory to inform the nature of data collected. Finally, we highlight the need to be responsive to stakeholders in order to enhance a study’s relevance.

## Background

### Performance-based incentives

Performance-based incentives (PBI) refer to a range of health system interventions that provide financial rewards based on the attainment and verification of predefined quantity and/or quality outputs [1–3]. As such, the approach represents a seismic shift in how health systems operate. Traditionally, facilities have been funded based on historical precedent or on metrics such as staffing levels, number of beds or the total population of the catchment area [4]. Performance-based approaches link incentives to desired outputs, thereby attempting to spark an entrepreneurial, autonomous spirit among providers. In the past 10 years, performance-based programs have proliferated across sub-Saharan Africa (SSA); as of 2013 at least three countries have national programs and 17 were conducting ongoing pilots [1]. A landscape analysis of programs that were initiated between 2008 and 2015 identified 32 programs across low and middle income countries (LMICs), including 25 programs across 21 countries in SSA specifically [5].

Initial evidence from pilots in low-income, SSA countries suggests that linking payment mechanisms to defined outputs can lead to increased service coverage and improved service quality. In Rwanda, results from two independent evaluations showed a positive impact of PBI on utilization for institutional deliveries, growth-monitoring consultations, and increased levels of perceived and technical quality of care (defined as compliance with national and international norms) [6, 7]. In the Democratic Republic of Congo, providing performance-based subsidies resulted in lower direct payments to health facilities for patients, who received comparable or higher quality services than patients receiving care in control facilities as measured via responses regarding patients’ overarching perceptions of quality, drug availability and respectful care toward patients by staff [8]. Despite these promising findings, a 2012 Cochrane review concluded that the existing evidence base is too thin to draw conclusions on the effects of performance-based interventions on the provision of health care and on health outcomes (whether related to treatment *or* prevention for maternal *or* child health) in LMICs [4].

While supporters of PBI claim that the approach can catalyze reforms and address structural problems of health services (including inefficiency and inequity) [9], reservations regarding PBI remain [4]. Three main criticisms include [10]: (1.) introducing financial incentives into a working environment characterized by a high degree of idealism may erode health workers’ intrinsic motivation (“crowding out”) [11]; (2.) the fact that PBI only addresses a limited range of indicators could lead to a neglect of non-remunerated aspects of work in favor of those that are remunerated (“gaming”) [12]; and (3.) there are considerable costs associated with implementing and monitoring PBI schemes [13].

Substantial knowledge gaps remain within the existing performance-based literature. In terms of program impact, there is a lack of research on intended and unintended consequences of such programs, limited understandings of patient and provider satisfaction with programs, and insufficient analysis of how PBI programming affects equity, resource use, organizational change and provider motivation [4]. Questions also remain as to how individual components of PBI schemes, both jointly and independently, affect quality of care outcomes [4, 14, 15]. In terms of program design, no evidence exists as to whether rewards not directly redistributed to health workers, but targeting exclusively facility improvements could result in increased access to quality services. In terms of program cost, limited evidence exists as to whether PBI schemes represent good value for money; we are aware of just one study that presents the costs of setting up and implementing a pay-for-performance program in a low-income context [16]. Finally, in terms of evaluation design, there is limited research that examines a PBI program in a holistic sense, by integrating elements of process, impact, and cost while relying on not only primary data, but also existing (secondary) data.

Our protocol represents an attempt to fill some of these knowledge and methodological gaps, by combining in a single study an assessment of the effects of a PBI program with an analysis of its implementation processes and with an economic evaluation, geared towards understanding PBI “value for money”. Our protocol is being implemented in Malawi external to the implementation of the SSDI-PBI program [17].

### Study setting

Malawi suffers from a heavy burden of HIV and communicable diseases (especially TB and Malaria) and, more recently, increases in non-communicable diseases (hypertension, diabetes and cancer) [18]. While the country has met several of its Millennium Development Goals (MDG) targets including those related to child mortality (MDG 4) and HIV and AIDS (MDG 6), other targets were not met including those related to maternal mortality (MDG 5) [19]. High rates of morbidity and mortality – particularly maternal mortality – have been linked to shortages in human resources for health, and inadequacies within facilities related to basic and essential infrastructure, management, support and services [20–22]. Healthcare delivery is largely centered around provision of an essential healthcare package (EHP) (including reproductive health services, child health services, as well as services related to the prevention, detection and management of infectious and non-communicable health problems) which is intended to be provided free of charge at point of use either in public facilities or in private not-for-profit facilities contracted by the Ministry of Health (MoH) [23]. Evidence indicates, however, that services included in the EHP are not as effectively available as they should be, thereby subjecting clients to substantial out-of-pocket expenditures [24–27].

Malawi’s MoH considers PBI a potential solution to the longstanding problem of inadequate service provision [28]. With financial support provided by the United States Agency for International Development (USAID), the Ministry of Health (MoH) of Malawi launched the Support for Service Delivery Integration (SSDI) Project in 2011 [29]. The objective of this initiative is to combine efforts of local and foreign organizations in order to strengthen the provision of EHP services [23].

Two SSDI sectors, SSDI-Systems and SSDI-Services, designed a PBI intervention that has been implemented in 17 facilities across three SSDI target districts (Chitipa, Nkhotakota, Mangochi) since 2014. Facilities were selected (non-randomly) based on minimum quotas related to equipment, infrastructure and personnel as deemed necessary to guarantee adequate EHP service delivery. Most facilities were also chosen based on their inclusion in a larger quality improvement program called “PQI” (for Performance Quality Improvement). PQI is based on the Standards-Based Management and Recognition (SBM-R) approach to quality improvement, which urges providers and staff consider the root causes and attainable solutions to address poor performance [30]. In this respect, the SSDI-PBI program builds upon an existing intervention that aims to improve provider performance and service delivery [30].

The SSDI-PBI program aims to increase access, utilization, and quality of EHP services by linking rewards to service utilization and quality indicators across a range of conditions and services. Utilization, or quantity, indicators focus on increasing total counts in terms of services across the maternal health continuum of care (during antenatal, delivery and postpartum periods), newborn and child health, and HIV and AIDS care and treatment (see Table 1 for a list of quantity indicators). Quality indicators emphasize improvements in the broader facility environment and in the nature of how care is provided across 13 service areas (see Table 2 for a list of quality dimensions). Quality assessments are complemented by a community component wherein a series of focus groups (a component of “community scorecards”) and exit interviews are conducted with clients to gauge satisfaction. SSDI-PBI rewards are comprised primarily of quantity and quality scores, with community scores serving as a source of potential bonus payments. Rewards are paid to facilities upon achievement of set targets, but the rewards can only be used toward facility improvements and cannot be partially redistributed in the form of performance bonuses to individual health workers, which is common under other performance-based schemes. Another defining characteristic of the rewarding system is that procurement at the facility level is managed through existing SSDI finance and procurement structures rather than through facility-based personnel. Rewarded funds are invested in previously determined service improvement activities or strategies outlined in annual business plans, which are developed by facility staff in collaboration with SSDI staff. These plans outline the activities, procurements or technical support that facility staff intend to prioritize as a means to improve care.

**Table 1:** Quantity Indicators used in SSDI-PBI

1.	Number of pregnant women starting antenatal care during the first trimester
2.	Number of women completing the four antenatal care visits
3.	Number of pregnant women receiving at least two doses of intermittent preventive therapy
4.	Number of births attended by skilled birth attendants (doctor, nurse or midwife)
5.	Number of 1-year-old children fully immunized
6.	Number of HIV-positive pregnant women who were initiated on antiretroviral therapy
7.	Number of HIV/AIDS cases screened for Tuberculosis
8.	Number of children receiving Vitamin A supplementation
9.	Number of clients counseled for family planning
10.	Number of couples tested for HIV during HIV testing and counseling services
11.	Number of infants born by HIV positive mothers tested for HIV
12.	Number of women who receive postnatal care after delivery by skilled health workers within seven days
13.	Number of pregnant women attending antenatal care receiving iron supplementation

**Table 2:** Quality Dimensions assessed in SSDI-PBI

1. General activities	
2. Follow-up assessment and HMIS	
3. Hygiene, environment, and sterilization	
4. Outpatient and inpatient consultation	
5. Maternity ward	
6. Antenatal consultation	
7. Family planning	
8. Vaccination and monitoring of newborns	
9. HIV/AIDS control	
10. Tuberculosis	
11. Laboratory	
12. Minor surgery	
13. Drug and commodity management	

### Study objective and theoretical model

The SSDI-PBI intervention is designed to influence the quantity and quality of service delivery, through the provision of incentives tied to facility performance and earmarked for reinvestment in facility infrastructure and equipment as outlined in facility-specific business plans. The SSDI-PBI intervention is therefore different from most PBI interventions implemented elsewhere in SSA in at least two fundamental respects [1, 31]. First, SSDI-PBI does not permit the provision of financial incentives in the form of salary top-ups or cash bonuses to individual health workers and/or service provider teams, thus it cannot be expected to directly increase health workers’ motivation (and, as a consequence, quality of health service provision) through increased remuneration. Furthermore, since the process of procuring goods, supplies and equipment acquired via PBI is managed largely by SSDI, facility or health worker-level autonomy in terms of acquiring additional resources appears to be restricted relative to other PBI schemes [1, 31].

### Theoretical model guiding this research

Despite the aforementioned factors, which could limit the effects of the intervention on health worker motivation and autonomy, it is plausible that additional funds generated by this PBI intervention could lead to increases in utilization and improvements in the quality of services via upgrades to both the physical and psychosocial working environment (in the form of structural improvements, the purchase and maintenance of currently lacking medical equipment and supplies). It also possible that the existence of the program could provide an opportunity – albeit with an element of pressure – for facility staff to better organize and outline their processes, to clarify performance and supervision expectations, and ultimately to galvanize one another in order to attain targets. Given that system weaknesses have often been linked to inadequate supplies [24–27], these improvements should almost inevitably result in better opportunities to provide clinical care. In turn, improved psycho-social working conditions are expected to enhance not only job satisfaction among health workers but also their motivation to enact clinical practices according to expected standards, thereby leading to improvements in the overall quality of care delivered. The positive relationship fostered by improvements in the working environment, in health worker satisfaction and motivation, and in health worker practices is likely to ultimately lead to higher client satisfaction, which could serve to further reinforce the working environment and health workers’ clinical and interpersonal performance.

The prevailing theory guiding performance-based literature is called principal agent theory [4]. The theory states that due to knowledge asymmetries that are inherent to the health encounter, it is necessary to devise novel strategies that align the goals and expectations of those who are principals (such as Ministries of Health) with the goals and expectations of agents (such as providers). Much like performance-based approaches, the theory urges that this bridging of goals can best happen via the alignment of rewards with targets [4, 32]. In other words, if an implementer or an institution (such as a Ministry of Health) makes its expectations more explicit by attaching a price to each service target, providers will adjust their behavior to respond to such a price signal.

While we appreciate the importance of goal setting and remuneration as a means to understand how and through which mechanisms PBI programs compel change, we find merit in expanding this view to consider the health encounter – and the environment within which health encounters occur – more broadly. To guide our conceptualization of the ways in which PBI interacts with the practices and routines of life in health facilities (with a particular influence on providers), we drew upon Social Cognitive Theory, a psychological model of behavior [33]. The theory highlights three key domains (“the triad”) that influence change: personal factors of an individual such as one’s sense of self-efficacy; behavioral factors in terms of an individual’s ability and experience enacting a set of behaviors; and environmental factors in terms of the availability of goods and supplies necessary to create change. Focusing on these three elements and the reciprocal determinism that exists among them, we focus our study on several facets of life in health facilities that are influenced in the context of PBI.

**Fig. 1:**
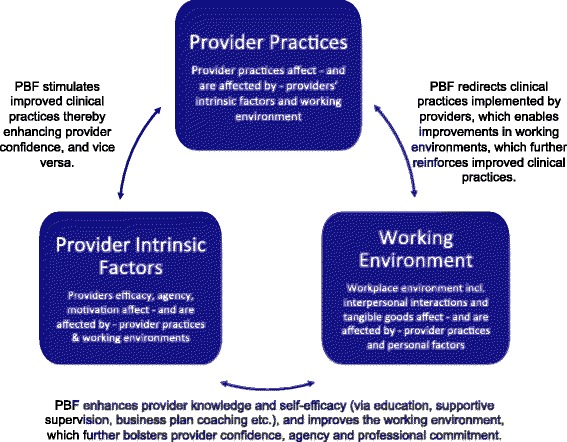
Reciprocity and Change within Facilities in the Context of PBF

We have adapted Social Cognitive Theory’s Cognition-Behavior-Environment triad to reflect our proposed triad of Provider’s Intrinsic Factors-Provider Practices-Working Environment. In Fig. 1, the uppermost box is labeled provider practices. We assert that PBI alters providers’ routines and behaviors; it can redirect certain clinical practices (including at the expense of other practices) and/or it can codify certain elements of clinical care (such as filling out partographs). This shift in clinical practices affects and is affected by providers’ internal cognition (see lower-left box labeled Provider Intrinsic Factors) as well as life in the broader facility (see lower-right box labeled Working Environment). In relation to the lower-left box, we hypothesize that PBI affects provider knowledge and self-efficacy (via PBI’s emphasis on education, supportive supervision and business plan coaching etc.), and that this could improve both the working environment and clinical practices. Finally, we argue that PBI programs lead to not only tangible changes in the health facility but also to changes in the social relationships and work routines in the health facility environment, which we refer to as the “Working Environment”. These changes could, in turn, spark improvements in the behaviors of providers and foster a sense of capacity, confidence and professional competence among individual providers. Taken as a whole, we use the highlighted theory as a conceptual springboard upon which to both understand the program and to design our study.

### Study objective

We are undertaking a combined impact and process evaluation, with an aim to assess the effect that the SSDI-PBI intervention produces on the work environment, on provider behavior (including service outputs) and on providers’ sense of knowledge, motivation and self-efficacy. Via a fidelity of implementation (FOI) assessment, we intend to look at intervention effects in light of contextual factors that shaped program implementation. Finally, we are also examining the cost of implementing the intervention in relation to outcomes produced. Our research questions are as follows:

1. Focusing on service provision, to what extent has the SSDI-PBI intervention produced changes in the quantity and quality of services provided? Which work environment changes can be attributed to PBI (i.e. availability of equipment, drugs, staff, training, supervision in respect to clinical performance)? What heterogeneity in effects can be observed across districts and facilities? To what extent have changes affected incentivized vs. non-incentivized services?
2. Focusing on providers specifically, how has the SSDI-PBI intervention changed motivation of health workers? Are changes in motivation reflected in changed attitudes or behavior at work?
3. Focusing on fidelity, to what extent has the SSDI-PBI intervention been implemented according to its original implementation plan? Which contextual factors have affected implementation, as defined in relation to acceptance and adoption of the intervention, at the various levels of service provision, including at health facility levels? Which contextual factors explain heterogeneity in implementation processes across districts and facilities?
4. Focusing on efficiency, what are the costs of implementing the SSDI-PBI intervention in relation to the outcomes produced? Is the economic burden of designing, implementing and managing the SSDI-PBI system worthwhile considering results achieved?

## Methods and design

### Overarching study design and methods

This study will rely on mixed-methods, applying both quantitative and qualitative methods of data collection and analysis (detailed in Table 3). The study will be partially prospective, collecting and analyzing primary and secondary data during the year of study implementation, and partially retrospective, relying on secondary data existing at the time leading up to and including the study launch. For a synopsis of methods and research questions employed see Table 3.

**Table 3:** Research questions, methodological approach, data collection activity

Research question divided by work package	Approach	Data collection	
		(a) Data collection instrument (b) Sampling unit (c) Data content	
		Quantitative	Qualitative^a^
1. Service Provision. How has the intervention affected quality of service provided and why?	Quant & Qual	a. Structured Checklist; Data extraction lists for (i) routine surveillance databases (HMIS) and (ii) Service Provision Assessment (at baseline)	a. IDI^a^& FGD^a^
		b. Health facility (intervention and control)	b. Clients, Community members
		c. Primary and Secondary data	c. Primary data
2. Provider Motivation. How has the intervention affected health worker motivation?	Quant & Qual	a. Health worker survey	a. IDI
		b. Health workers in intervention and control	b. Health workers in intervention facilities
		c. Primary and secondary data	c. Primary data
3. Fidelity of Implementation. How has the intervention aligned with intended design, and what factors have affected this?	Qual	--	a. IDI & Document Review
			b. IDIs with MoH, Funders, PBI desk officers, DHMTs, SSDI employees, health workers in Intervention Facilities; Document Review of implementation planning and monitoring material
			c. Primary and Secondary data
4. Costing. What are the costs of implementing the intervention in relation to outcomes produced?	Quant	a. Data Extraction	--
		b. Implementer materials (SSDI costing data)	
		c. Secondary data	

^a^Methods acronyms: *IDI* is in-depth interview, *FGD* is focus group discussion

### Changes in facility routines and Provider’s lives

#### PBI’s impact on service provision and health worker behavior

Drawing on a mixed-methods approach, this component will focus on changes produced by the SSDI-PBI intervention on quantity and quality of the health services provided in relation to measured changes in the work environment. Following our theoretical model, changes in physical and psychosocial work environment are expected to positively affect EHP service delivery. Specifically, we expect to observe changes in the quality of care of targeted services.

Quality of care measures will include measures of service input, process, and output elements, such as the availability of functional equipment, stock-outs of essential supplies and medicines, procurement and maintenance procedures at both the individual service and the facility level, human resource availability, client satisfaction with essential service components, and service coverage.

Given our explicit ambition to work with a design closer to implementation by building on health information that is collected on a routine basis, most of the data used in this study component will stem from secondary sources (health management information system (HMIS), service provision assessment (SPA), SSDI baseline data, as well as SSDI monitoring and evaluation data). Additional information collected through direct facility inventories and health worker surveys will be used to further enrich the data sources for this component.

This portion of the study will use mixed methods, collecting the above quantitative but also qualitative data in parallel with an aim to explore different facets of the same research question. The quantitative component will adopt a controlled time-series design, including controls that are matched to be comparable in terms of facility type, zone (or district, if feasible), distance to a main road, and PQI intervention status. The inclusion of controls is meant to minimize potential time-dependent biases. For all selected indicators, we will therefore collect information going as far back as 12–24 months prior to and 12–15 months following the PBI program’s start. The qualitative portion of this component will be used to examine clients’ perception of service quality over the course of and in response to the PBI intervention. Qualitative methods of data collection will include IDIs with clients (i.e. service users) and FGDs with community members (including those who can convey community-held knowledge, attitudes and practices in relation to the health facility).

#### PBI’s effect on health workers’ motivation

Drawing on mixed methods, this component will focus on health workers’ perceptions, satisfaction and motivation in relation to the implementation of the SSDI-PBI intervention. Specifically, we will assess whether and how changes in the working environment are perceived by health workers, and how these changes might have resulted in changes in motivation to provide high quality and quantity care. The qualitative approach will entail a series of IDIs with selected health workers from selected intervention health facilities, taking a retrospective approach of asking them to recall their perceptions of changes that have taken place in the implementation period, relating them to specific aspects of the intervention, and explaining pathways of change. We will conduct interviews only in intervention facilities with an aim of interviewing at least two providers per facility. We will also collect quantitative survey data that will assess perceptions of change in the working environment in both control and intervention facilities.

### The process evaluation component

Drawing on qualitative methods, the process evaluation will focus on fidelity of implementation (FOI), and consider adherence (defined in terms of content, schedule, and coverage) to the original intervention model and the contextual factors that mediated and affected adherence [34, 35]. This analysis will explore to what extent *in itinere* modifications - inevitable when concerned stakeholders are responding to and implementing a given intervention - hinder or enhance the effectiveness of the intervention itself. The process evaluation component will allow us: to delineate to what extent the SSDI-PBI intervention was delivered as initially planned and to explore heterogeneity in implementation processes across districts and facilities; to understand how the various stakeholders responded to the intervention and acted to modify, for better or for worse, its content; to identify contextual elements that affected the implementation of the SSDI-PBI intervention and to what extent these elements can explain heterogeneity in outcomes across districts and facilities.

Within this component we will pay particular attention to the implementation processes related to: the verification and counter-verification system, to understand its suitability and effectiveness within the framework of the SSDI-PBI intervention; the facility management structures and the interaction with the SSDI team in charge of the facility business plans, to understand the process of receiving and re-investing the performance rewards and the potential for “gaming” induced by these structures; and the role that district management teams play in relation to supervision, human resource allocation, and distribution of equipment and commodities, to identify potential changes in behavior in favor of incentivized facilities and services.

This portion of the study will rely on qualitative methods, conducted using a prospective approach. In line with standard practice in process evaluation [36] and with our own work in similar settings [37, 38], we will begin our work by convening a workshop with the SSDI-PBI core design and implementation team to develop a shared Theory of Intervention (TOI) and to identify the core 20–25 activities which fit this TOI, and are key to the intervention success from a theoretical point of view. Qualitative data will be collected to examine contextual factors that mediated the implementation and as such affected fidelity, and to explore how the various stakeholders responded to the intervention through adaptation and adoption. By “stakeholders” we are referring to anyone involved in the design and implementation of the intervention (i.e. the funding agency and its implementing partners, Ministry of Health (MoH) directorates, district management teams, PBI desk officers, as well as at least one health worker from each concerned facility). Sampling will be done via the snowball method wherein the study team will first be introduced by the funding agency and key programmatic personnel to those engaged in the SSDI-PBI program. This will assist the research team in identifying initial respondents. These respondents will be asked to assist in the identification of other respondents, who could facilitate an understanding of the themes and issues raised in the interview or related to the program generally.

### The costing component

The main objective of this component is to evaluate costs of the SSDI-PBI scheme to provide insights on the value for money associated with the intervention. The costs associated with PBI include the following cost categories: a.) start-up costs (design of program, training, initial dissemination); b.) ongoing management (including verification and counter-verification); c.) the cost of incentives themselves. These costs are incurred at the level of the Ministry of Health, its development partners (USAID), and the implementing agency (SSDI). The cost analysis will allow estimating the incidence of different cost categories, accruing the total costs of PBI. More specifically, we will be able to compare the total costs of start-up, implementation and management of the PBI scheme to the costs of financial incentives. For the majority of cost components, costs will be evaluated using the micro-costing approach, which requires that for each cost category, quantity of resources is identified and then multiplied by its unit costs. To do this we will rely heavily on support from the implementation team, asking to access their financial and cost management data. We will also request lists of participants who were engaged in key implementation activities (and approximate salary ranges across cadres of employment) in order to account for an allocation of staff time toward PBI activities in the event that time investment was not paid for.

### Overarching analysis & data protection

The ultimate aim of this research is to assess the effect that the SSDI-PBI intervention produces on the quantity and quality of care in light of the intervention implementation processes and at what cost. While the work is divided into components to ease data collection and analysis, the ultimate aim of this study is to triangulate and integrate information across components and data sources in order to address the overall study objective, and to provide a more nuanced interpretive analysis. Lead researchers across study components will convene to discuss and share findings upon completion of analysis of a given component.

### Quantitative data analysis

Quantitative data will be analyzed using Stata. Our analytical approach will largely rely on a controlled time-series analysis, building on data from 17 intervention and 17 control facilities. As such, the actual sample size will be 34. For each of the quantitative outcome indicators, we will include monthly data from the HMIS for the period Sept 2013 to Dec 2015 (or 12–24 months prior to and 12–15 months following PBI start). This will generate a total of 28 observation points for each of the 34 facilities included in the study. For each service provision indicator, we will compare developments over time across intervention and control facilities. Given that we could not assess actual data availability and completeness prior to data collection, we will do ex-post power calculations. We will assess the impact of the intervention on targeted service provision indicators using an interrupted time-series model with independent controls. In addition, given the availability of two single time points, we will rely on difference in differences modeling to assess changes in quality of care, using for example changes in staff numbers and qualification, availability of equipment and supply as proxies. This analytical component will draw upon primary data that will be collected in intervention and control facilities, as well as SPA data collected in January 2014, which will serve as a pseudo-baseline.

### Qualitative data analysis

All qualitative interviews will be tape-recorded, transcribed and translated into English. Qualitative data will be analyzed using a hybrid approach. We will first identify themes from a selection of rich and nuanced transcripts – an inductive approach [39]. We will later apply this template of codes to the remaining transcripts through a deductive approach [40]. We will adopt in-vivo coding (using NVivo 10), with codes, categories, and themes emerging as we proceed through the data, although the initial coding process will be guided by the research questions. We will apply data triangulation as we will compare information across data sources to capture multiple perspectives of the same research question, and analyst triangulation as at least two researchers will code and interpret each set of data.

## Discussion

PBI programs are complex and questions remain in terms of the potential for such programs to improve the quality of care and spark reductions in morbidity and mortality. The aim of SSDI-PBI in Malawi is to increase service utilization and stimulate better quality of care by linking payments to strategies aimed at improving providers’ working environments and in doing so, empowering providers with the means to provide care of an adequate standard.

The overarching purpose of this study is to contribute robust evidence to a growing body of literature examining the process, impact and cost-effectiveness of PBI programs in SSA. We also aim to generate knowledge regarding how programs such as SSDI-PBI are woven into the health system, how they are perceived by patients and providers alike, and to what extent they are cost-effective. We view this research as particularly useful because it examines a non-conventional PBI model, which does not entail monetary incentives or financial autonomy at the facility level. Such a model has not been studied in the literature.

This study design was conceptualized with a relatively small budget and a relatively short study period (approximately one year) to rapidly meet the need of informing the implementation of the intervention. Given the broad scope of the PBI intervention in respect to the range of services being incentivized, time and funding constraints do not allow for a detailed assessment of cost, nor is it feasible to undertake time or labor intensive endeavors such as community-based household surveys to assess health-seeking behavior, or direct observations to assess quality of care processes. In respect to producing robust effect measures, the main limitation of this study design is that it had to be adapted to a non-randomized intervention. Given that study districts and intervention facilities were chosen by the MoH based on political and logistical considerations rather than a random selection of health facilities and districts, selection bias was introduced. Furthermore, our study sample is limited to the relatively small scope of the intervention (17 health facilities), potentially resulting in low statistical power. As a consequence, we might not be able to demonstrate statistical significance of observed impacts. Finally, the fact that the start of the PBI program is not aligned with the external evaluation research further restricted the design, as baseline information on the performance of essential health service provision could not be based on primary data collection on sets of indicators directly linked to specific outcome measures. As a result, different quasi- and non-experimental study approaches and a heavy reliance on secondary data sources was required to address potential threats to validity.

In terms of contending with the aforementioned limitations, our evaluation team seeks to be nimble and resourceful. We are drawing from multiple methods within both quantitative and qualitative approaches. Quantitatively, we collect primary data related to infrastructure and equipment, staff numbers and trainings, as well as health worker perceptions. Qualitatively, we directly collect IDI and FGD data across a broad range of respondents at facility, community and policymaker levels thereby inhibiting the domination of one single respondent type. We are also undertaking triangulation to identify points of convergence and divergence in the data. This study draws upon a substantial amount of secondary data. The utilization of secondary data, while imperfect, can present a means to capitalize on existing data thereby reducing costs while contending with time constraints. Furthermore, secondary data presents a means to overcome the absence of an evaluator-initiated baseline survey, to capture trends over time (in the case of HMIS data, specifically), and to fulfill a tenet of meaningful implementation research [41]. While having a short turnaround period poses limitations in terms of research, we also view it as an opportunity to generate knowledge and to inform implementation partners in a timely fashion—even as decisions are being made regarding how to expand an intervention.

This study also seeks to draw upon a theory as a means to inform the nature of the research and to guide the research team in terms of sampling and data analysis. We view the development of theory as an essential though under-emphasized concept within PBI literature. This evaluation places an emphasis on the health facility (inclusive of the staff, infrastructure and equipment therein), which the study team and implementing team have determined represents a meaningful starting point for an examination of PBI (given the manner in which the program compels tremendous shifts in terms of how, when, how often and at what cost providers are capable and expected to provide care). Furthermore, the actions of providers and the nature and quality of their engagement with patients are critical factors in determining whether and to what degree morbidity and mortality are ultimately reduced. While our theory emphasizes the effect of PBI within the health facility, we see several points of entry to inform future consideration of the theoretical underpinnings of PBI. For example, PBI lays the foundation for national level policies and priorities that ultimately redirect practices at zonal, regional, district and community levels. On an interpersonal level, PBI affects how patients and providers interact with and perceive one another (and possibly how the parties perceive the health system more generally). Given the web of entry points wherein one could consider theoretical underpinnings of PBI, it is challenging to focus on a particular sphere or set of indicators, but we view an expansion of theory-driven research as necessary in guiding future research in this field.

Our study design contributes to the evaluation literature by providing an example of how sound implementation research can be done despite time and budget constraints as well as constraints due to the size of the intervention. We view this contribution as crucial particularly as PBI programs continue to expand and ministries of health and finance -- as well as funders and program implementers -- are faced with decisions regarding whether or how programs should be adapted or expanded and at what cost. We view a more well-rounded examination as a powerful tool in not only guiding programmatic and policymaker decisions, but also as a means to guide fellow researchers as they consider how to evaluate health programs in a comprehensive, affordable, meaningful and timely manner.

## Abbreviations

CoM, college of medicine (of Malawi); EHP, essential health package; FGD, focus group discussion; FOI, fidelity of implementation; HMIS, health management information system; IDI, in-depth interview; MDG, millennium development goal; MoH, ministry of health; PBI, performance-based incentives; SPA, service provision assessment; SSA, sub-saharan africa; SSDI, support for service delivery integration; TOI, theory of intervention; USAID, United States Agency for International Development
